# Predictive coding and neurocomputational psychiatry: a mechanistic framework for understanding mental disorders

**DOI:** 10.3389/fpsyt.2025.1713833

**Published:** 2026-01-07

**Authors:** Alexander D. Shaw, Rachael L. Sumner, Lioba C. S. Berndt

**Affiliations:** 1 Department of Psychology, Faculty of Health & Life Sciences, University of Exeter, Exeter, United Kingdom; 2 Department of Biomedicine and Medical Diagnostics, Auckland University of Technology, Auckland, New Zealand; 3 School of Physiology, Pharmacology and Neuroscience, University of Bristol, Bristol, United Kingdom

**Keywords:** active inference, free energy, mechanistic framework, neurocomputational psychiatry, predictive coding

## Abstract

Predictive coding offers a powerful computational framework for understanding brain function and psychiatric disorders at a mechanistic level. This perspective synthesizes advances in computational psychiatry, proposing that mental disorders can be conceptualized as specific alterations in the brain’s predictive inference machinery. We first outline the theoretical foundations of predictive coding, including Bayesian inference, free-energy minimization, and neural population dynamics, illustrating how these abstract computational principles map onto specific neural circuits and biophysical mechanisms. We then argue that diverse psychiatric conditions can be understood within this unified framework. Taken together, these links between theory, generative models and empirical data suggest a route by which predictive coding might be rendered a testable, modifiable, falsifiable construct within biological psychiatry. Beyond offering conceptual clarity, this framework has significant clinical implications, including the development of mechanistic biomarkers, personalized treatment approaches based on computational phenotypes, and novel therapeutic interventions targeting specific inferential abnormalities. By grounding psychiatric symptoms in aberrant predictive processes implemented in neural circuitry, this approach promises a more mechanistic understanding of mental disorders and a path toward more targeted, effective interventions.

## 1 Introduction

Traditional psychiatry has long relied on symptom-based classifications of mental illness, with limited insight into underlying brain mechanisms. Computational psychiatry offers a new paradigm (1–5): by integrating mathematical models with neurobiology, it seeks to explain psychiatric phenomena in terms of aberrant brain computations (6). Among various modelling approaches, predictive coding has emerged as a compelling unifying theory for brain function (7, 8). In essence, predictive coding treats the brain as an inference machine that continually generates and updates predictions about sensory inputs via hierarchical message passing. Closely related, but conceptually distinct (9), is the Free Energy Principle and its formulation in terms of active inference, which extend predictive coding from perceptual inference to action and policy selection (10). In this article we focus primarily on predictive coding as an algorithmic and neurobiological account of hierarchical inference, while drawing on active inference where it clarifies the role of behaviour, policy selection, and intervention. This family of frameworks provides a normative (Bayesian) account of perception and action, in which the brain seeks to minimise surprise or “free energy” by aligning its internal model with the outside world (11). Crucially, this perspective offers a plausible account of neuronal computation in cortical circuits: hierarchical networks of neurons are thought to exchange top-down predictions and bottom-up prediction errors to achieve efficient information processing.

At the same time, predictive coding provides a principled way to think about mental illness. Many psychiatric symptoms can be interpreted as errors of inference - that is, failures in the predictive coding machinery (12). For example, hallucinations and delusions in psychosis may result from placing too much weight on prior beliefs (or not enough on sensory evidence) - a computationally precise deficit that could serve as a quantitative biomarker for psychosis subtypes. Conversely, autistic perception may stem from overly weak prior expectations leading to sensory overload, representing a fundamentally different computational phenotype. This review will examine how major psychiatric conditions can be framed in terms of aberrant predictive coding, linking computational deviations to clinical phenomena.

Finally, predictive coding is especially attractive because it maps onto neural circuits. The abstract variables of predictive models (predictions, prediction errors, precision weights) can be associated with specific neuron populations and connections in cortical microcircuits. These concrete mappings mean that predictive coding hypotheses are testable with neurobiological data (11). Using neural mass models and Dynamic Causal Modelling (DCM), researchers can design generative models of brain activity and compare them to recordings (M/EEG, fMRI) to quantify these computational parameters in individual patients, moving toward personalized computational phenotyping in psychiatry. Since DCM was first introduced in 2003 (13), the models themselves are being refined, expanded and tested.

In what follows, we outline the current state of the art of the predictive coding framework and its neural implementations, then explore how it illuminates psychiatric disorders, and finally discuss the broader implications for diagnosis and treatment in psychiatry.

## 2 Theoretical foundations of predictive coding in the brain

### 2.1 Brain as an inference machine – bayesian reasoning and dynamics

A central premise of predictive coding (and related theories like the Bayesian brain hypothesis) is that the brain performs some form of Bayesian inference (14). The brain maintains internal generative models that produce predictions about sensory inputs, and it updates these models when predictions fail (8, 10). Mathematically, we can describe the brain’s state dynamics and outputs with differential equations (13). For example, a simple state-space model of neural activity can be written as:

(1) $$\frac{dx}{dt}=f(x,\;u,\;P),\;y=g(x,\;P),$$

where x represents internal neural states (variables), u external inputs, P model parameters, and y observable outputs (e.g. neural signals). Here, f encapsulates how neural states evolve over time (governed by physiology), and g maps internalstates to observed signals. In practice, P denotes the collection of parameters governing both f and g, with different subsets typically influencing the dynamics and the observation mapping, respectively.

In healthy function, these dynamics settle into stable regimes (attractors) that support adaptive perceptions and behaviours. In contrast, psychiatric disorders may correspond to pathological attractor states in this system – for instance, depression might involve a neural state getting “stuck” in a low-firing, inflexible regime. By formulating such models and fitting them to patient data (e.g. M/EEG), we can infer which physiological parameters ( P) are altered in illness (for example, synaptic gain or connectivity) and thereby gain mechanistic insight (13). From this perspective, predictive-coding updates (changes in $\hat{\mu }$ driven by precision-weighted prediction errors) can be viewed as shaping the flow field f(x) in Equation 1; describing psychopathology in terms of ‘pathological attractors’ is therefore a dynamical-systems re-expression of the same underlying inferential process, rather than a distinct mechanism.

### 2.2 Predictive coding and free-energy minimisation

Predictive coding extends the above by positing a specific computational strategy for the brain: minimise the error between expected and actual inputs (15, 16). In a predictive coding scheme, higher brain regions send predictions (top-down signals) about lower-level activity, and lower regions compute prediction errors (differences between what was predicted and what is actually sensed) to send back upward. The brain then adjusts its internal states to reduce these errors. A simple formulation of predictive coding (Equation 2) is:

(2) $$\text{Δ}\hat{\mu }=\eta \epsilon ,$$

where $\text{Δ}\hat{\mu }$ denotes the change in the current estimate over a small time-step or iteration, $\hat{\mu }$ is the brain’s current prediction (or estimate of a latent cause), ϵ is the prediction error (difference between observed input y and the predicted input $\hat{y}$), and η is a learning rate.

In other words, the estimate is adjusted in proportion to the error signal. This can be seen as a gradient ascent step on an implicit log-likelihood or a descent on “surprise.” By iteratively refining its predictions in this manner, the brain approaches a state that maximises model evidence and minimises surprise (or variational free energy). Notably, this framework is formally equivalent to Bayesian inference: the brain’s updated estimate $\hat{\mu }$ comes to approximate the posterior belief that combines prior expectation with (sensory) likelihood. The Free Energy Principle generalises this idea, proposing that neural dynamics minimise a free- energy bound on the discrepancy between the brain’s model and sensory data (10). This theoretical principle bridges computation and neurobiology: under certain assumptions (e.g. Gaussian noise), it yields biologically plausible rules for synaptic updates that implement Bayesian belief updating in neural circuits. Together, these formulations provide a perceptual account of free-energy minimisation. However, the free-energy principle is broader than perceptual inference alone: it also implies that organisms can minimise uncertainty through action, actively sampling the environment to reduce ambiguity and confirm or disconfirm their predictions, as formalised under active inference (17).

A related way to view predictive coding is through the basic structure of Bayesian inference. Posterior beliefs arise from combining prior (P) expectations about a latent state with the likelihood of observing the current sensory input (Equation 3).

(3) Posterior∝P(observation|state)×P(state)

The relative precision of the prior and the likelihood determines which source of information shapes the posterior more strongly. High-precision priors bias inference toward expectations, whereas reliable (high precision) sensory evidence gives greater weight to incoming observations.

### 2.3 Neural populations and mean-field models

Brain networks consist of large populations of neurons making detailed single-cell modeling computationally prohibitive. To connect microscopic neural activity with macroscopic brain signals (e.g. M/EEG or fMRI), predictive coding models often invoke a mean-field or neural mass approximation (18). Rather than track every neuron, one tracks the average activity <*χ*> of a population, along with summary statistics of variability. For example, a mean-field equation might describe the evolution of the population firing rate, while a second equation captures the variance or correlations within the population. Such population models are integral to implementations of predictive coding in the cortex: they allow one to treat an entire cortical column or region as a unit that sends and receives prediction/error signals (19). Importantly, the hierarchical organisation – populations organised in layers and areas – naturally lends itself to the hierarchical Bayesian structure of predictive coding (20). Eachlevelofthehierarchydealswithadifferentscaleofrepresentation, and population dynamics at that level encode predictions or errors about that content. By adjusting a few key parameters (like the gain of neuronal populations that carry error signals), these models can simulate how precision (confidence) is encoded and modulated in the brain (21, 22). This provides a way to link neurotransmitter systems (e.g. NMDA, dopamine or serotonin, which affect synaptic gain) to computational quantities in predictive coding (like precision-weighting of prediction errors). Such connections are central to understanding conditions in this framework where altered neuromodulatory systems may directly impact precision-weighting and inference.

### 2.4 Biophysical generative models – Hodgkin–Huxley and beyond

An attractive aspect of predictive coding theory is that it can be grounded in detailed neurobiology. The function f(x) in our state equations (Equation 1) can be specified using well-established biophysical models of neurons. A classic example is the conductance-based Hodgkin–Huxley model (23), which describes how a neuron’s membrane voltage *V* evolves over time due to ionic currents (Equation 4). In a Hodgkin–Huxley formulation:

(4) $$C_{m}\frac{dV}{dt}=g_{\text{Na}}m^{3}h(E_{\text{Na}}-V)+g_{\text{K}}n^{4}(E_{\text{K}}-V)+g_{L}(E_{L}-V),$$

where $C_{m}$ is the membrane capacitance, $g_{\text{Na}},g_{\text{K}},g_{L}$ are the maximal conductances of sodium, potassium, and leak channels, $E_{\text{Na}},E_{\text{K}},E_{L}$ are their reversal (Nernst) potentials, and m,h,n are gating variables that evolve according to their own dynamics. This detailed equation is a concrete instantiation of the function f(x) governing neuronal dynamics, and can be easily extended to include a range of neurotransmitter systems (for example, adding calcium channels and a voltage gate to model NMDA (24);).

While such biophysical complexity is often simplified in higher-level models, it reminds us that any computational theory like predictive coding ultimately must respect the laws of neurophysiology. To bridge single-neuron dynamics with neural population behavior, these biophysical principles can be incorporated into mean-field models, creating conductance-based neural masses that maintain biological realism while allowing scalability. Indeed, one can build generative models that incorporate known physiology (e.g. receptor kinetics, membrane time constants) and then invert those models to explain observed neural data. Computational psychiatry studies have done exactly this – for instance, using conductance-based neural mass models to infer synaptic changes in disorders like schizophrenia from M/EEG recordings (25, 26).

## 3 Neuronal circuitry of predictive coding

**Figure 1:**
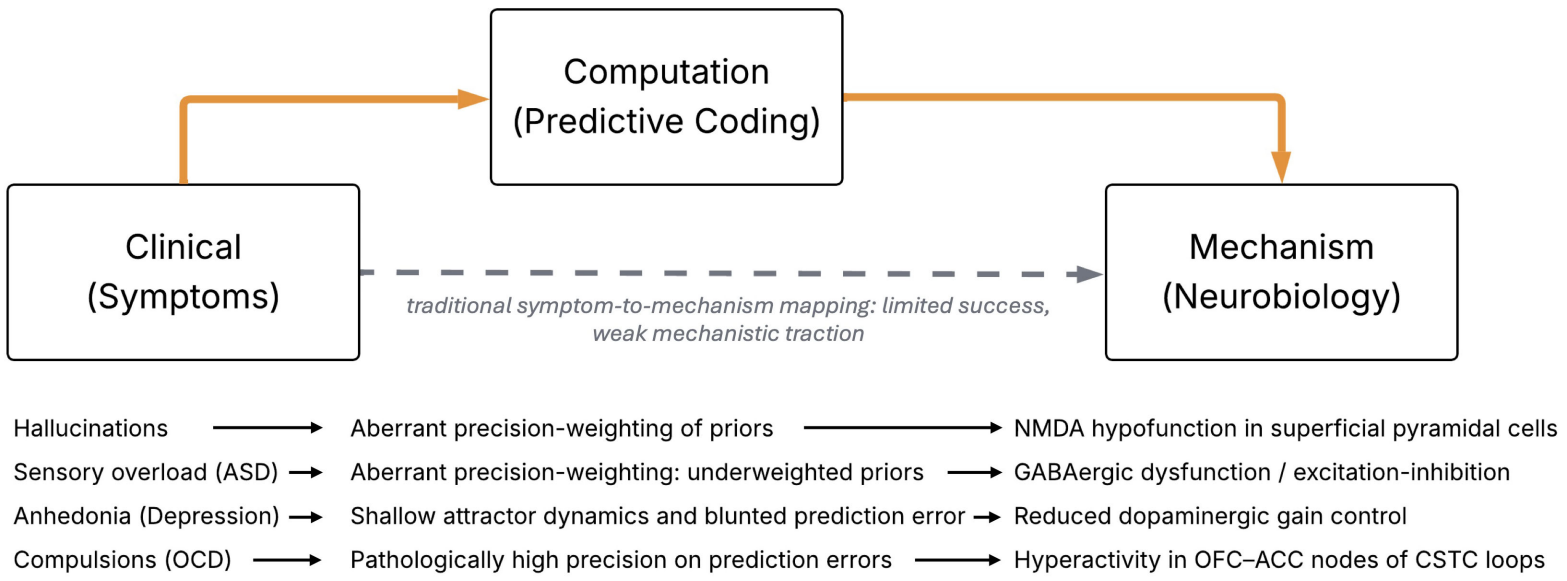
Computational models, here exemplified by predictive coding, as a bridge between psychiatric symptoms and neurobiological mechanisms. Traditional approaches (grey dashed line) attempt to map clinical phenomena directly onto biological substrates, often yielding limited mechanistic insight. Predictive coding provides a principled intermediate layer (orange arrows), allowing symptoms to be reframed as computational inference failures-such as aberrant precision-weighting or dysfunctional attractor dynamics-which can in turn be grounded in specific circuit-level or receptor-level pathophysiology. Other computational frameworks (e.g. reinforcement-learning or control-theoretic models) can also occupy this intermediate level. Examples illustrate this tri-level mapping for hallucinations, sensory overload, anhedonia, and compulsions.

Building on the theoretical foundations discussed above, we now consider how predictive coding might be implemented in actual brain circuitry. A growing body of work suggests that canonical cortical circuits – the repeating layered networks in cortex – are well-suited to implement the hypothesised message-passing of predictions and errors. In a hierarchical predictive coding model, every cortical area (or layer) has units that encode the current prediction of some features and units that encode the prediction error (the unexplained residual). Physiologically, a plausible mapping is that deep-layer pyramidal neurons carry top-down predictions to lower areas, while superficial-layer pyramidal neurons carry forward prediction errors to higher areas (**Figure 1**). In this hierarchical architecture, predictions flow downward and prediction errors flow upward, but the influence of either pathway depends on their estimated precision. Precision acts as a weighting term that determines whether inference is dominated by sensory evidence or by prior expectations, making it a central quantity in predictive coding accounts of psychopathology. Here, precision refers to the estimated reliability of prediction errors within the generative model; this differs from metacognitive confidence, which reflects a higher-order judgement about one’s own decisions (27).

**Figure 2:**
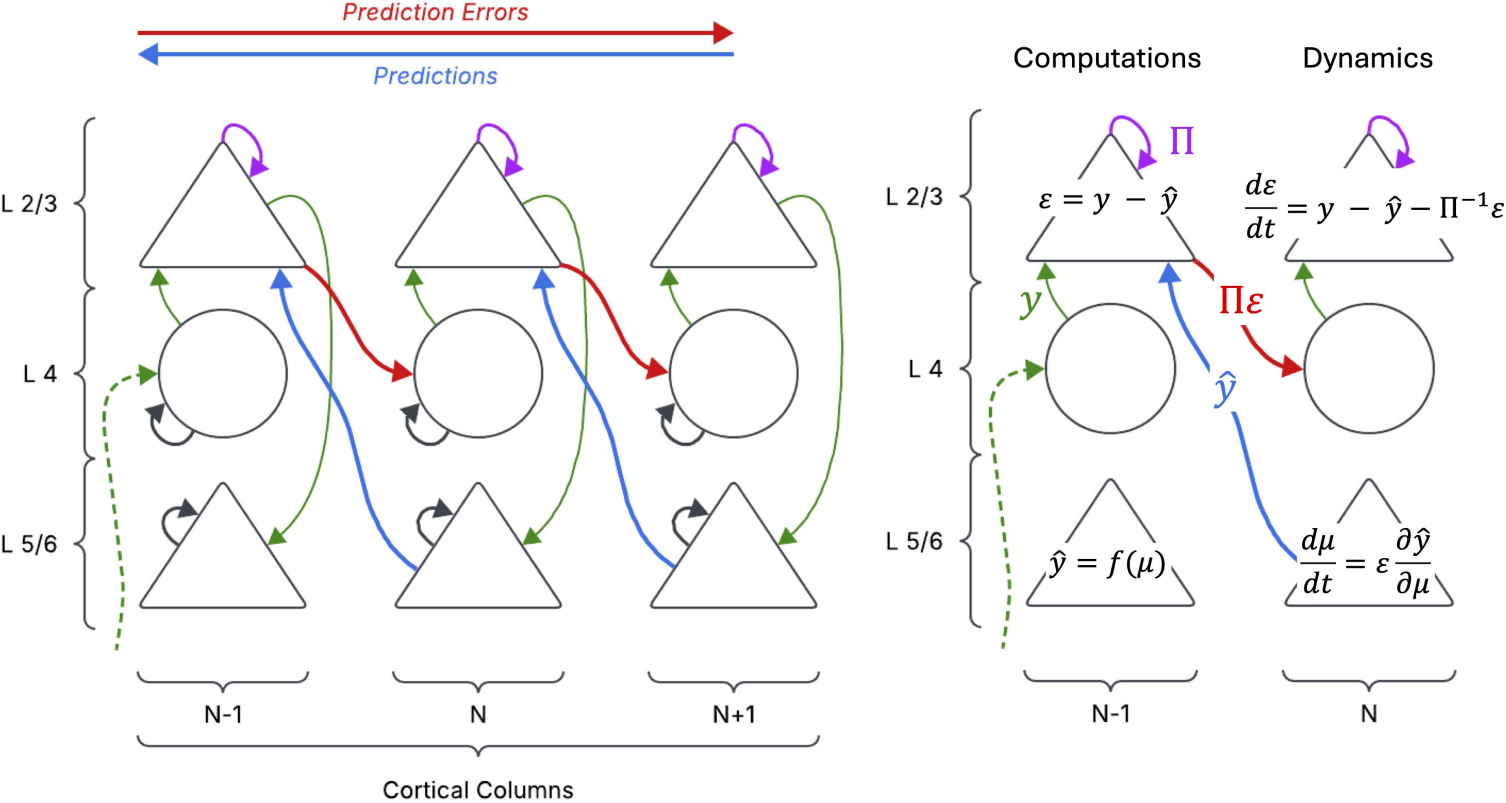
Schematic of hierarchical predictive coding across cortical levels. Left panel: Higher areas (N+1) send predicted signals (feedback, blue arrows) to lower sensory areas (N), forming empirical priors for incoming data. At the lower level, the difference between actual input (green arrow) and the top-down prediction constitutes a prediction error. This error is weighted (purple arrow) and passed forward (feedforward) to update the higher-level representation (red arrow) in the form of a ‘precision weighted prediction error’. Right panel: The encoding of predictions and prediction errors plausibly takes place in deep (L5/6) and superficial (L2/3) pyramidal populations, respectively. Furthermore, since this process is dynamical (rather than fixed) these computations can be formulated as differential equations (far right).

Supporting this model, neuroanatomical studies have found a remarkable correspondence between the connectivity of cortical microcircuits and the connections implied by predictive coding theories (20). Specifically, the laminar patterns of feedforward vs. feedback projections align with the idea that separate neuronal populations send predictions downward and errors upward in the hierarchy. For example, feed-forward projections originate mainly from superficial layers (L2/3) and target middle-layer (L4) neurons in the next cortical area, whereas feedback projections originate from deep layers (L5/6) and target superficial layer neurons in the preceding area (28). This matches the predictive coding requirement for distinct streams of information flow. A detailed model by Bastos and colleagues (20) formalised this, assigning biophysical neuron models to the roles of error units and prediction units and demonstrating consistency with observed cortical beta and gamma rhythms. Inhibitory interneurons also play crucial roles in this framework, potentially controlling precision-weighting through their modulatory effects on pyramidal cell activity. Moreover, the involvement of the thalamus is often interpreted in predictive coding terms: the thalamus might help compare cortical predictions with incoming signals, or gate the precision of ascending sensory data (29) (**Figure 2**). Dysfunctions in thalamo-cortical loops are indeed implicated in disorders like schizophrenia and OCD, consistent with predictive coding abnormalities (e.g. thalamic filter failure leading to sensory overload or intrusive errors) (2).

Because predictive coding neatly maps onto specific circuits, we can use data-driven modelling to test hypotheses about those circuits. DCM is a prominent approach that evaluates network models against measured brain signals (30). In practice, most M/EEG and fMRI DCM studies operate at the level of interacting 153 cortical regions or laminar-population models rather than individual neurons. Neural mass DCM captures population-averaged dynamics within regions, whereas conductance-based or laminar DCM can resolve layer-specific or receptor-specific mechanisms within those populations. In DCM, one posits a circuit model (with directed connections, layers, etc.), and uses Bayesian inference to estimate the connection strengths that best explain the data for different groups or conditions. Predictive coding provides guidance for constructing such models (e.g. which connections should change under certain tasks or pathologies). A striking example comes from a DCM study on people with schizophrenia performing a perception task (6). The modelling results showed that compared to healthy subjects, schizophrenia patients had markedly reduced backward connectivity from frontal cortex to visual cortex, and moreover, unlike controls, they failed to increase this top-down connectivity when stimuli became predictable. In other words, the normal adaptive tuning of cortical feedback based on predictability was absent in schizophrenia – exactly what one would expect if the brain’s predictive coding machinery (which relies on adjusting top-down signals) was impaired. This provides a concrete example of how predictive-coding-inspired generative models can be fitted to empirical neuroimaging data to test mechanistic hypotheses about altered connectivity in psychiatric populations. By examining circuit parameters estimated via DCM, we obtain evidence that “precision-weighting of prediction errors is deficient” or “top-down predictions are underutilised” in a given disorder.

## 4 Predictive coding and psychiatric disorders

Using the predictive coding lens, we can reinterpret several major psychiatric conditions as specific forms of inference gone awry (31). In each case, symptoms are linked to particular disturbances in how predictions, prediction errors, or their precision weights are handled in the brain (32). Crucially, because of the mapping between predictive coding and cortical wiring, proposed changes in predictive coding represent testable hypotheses of pathophysiology with biologically meaningful parameters (12, 33). Below, we review key examples and supporting evidence.

### 4.1 Schizophrenia: aberrant precision-weighting of priors

Schizophrenia has been hypothesised to result from an imbalance in the brain’s handling of prediction errors and priors. In healthy perception, the brain assigns an optimal precision (or confidence) to sensory evidence relative to prior beliefs, so that neither hallucinations (overly strong priors) nor confusion (overly strong sensory noise) occurs. In schizophrenia, this balance appears to be disrupted: individuals may place too much weight on internally generated predictions (priors) and not enough on external sensory input (2). In predictive coding terms, there is an aberrant precision-weighting such that top-down signals are afforded inappropriately high precision relative to bottom-up signals. Formally, one can think of the prediction error term:

(5) $$\epsilon =(y-\hat{y})$$

being under-weighted, or conversely the prior’s influence being over-weighted, due to mis-tuned precision (Π) on error neurons. Here, “dysfunction” refers to systematic biases in these precision parameters and related synaptic gains rather than a single deficit which alter how beliefs are updated from sensory evidence.

This idea helps explain classic positive symptoms: delusions can be seen as unfounded beliefs that persist because contradictory sensory evidence (prediction errors Equation 5) is not given enough weight to overturn them. Hallucinations, likewise, could result from internally generated representations (predictions from higher cortex) intruding on perception because the brain is overly biased toward expecting its own hypothesis rather than the actual input (34). In computational simulations, reducing the “precision of priors relative to sensory evidence” indeed produces hallucination-like phenomena. Related predictive-processing accounts also link precision abnormalities to negative symptoms. Rather than overweighted priors in perception, negative symptoms are thought to involve blunted or imprecise reward-related prediction errors that fail to update future-oriented value expectations. Recent altered-anticipation and anticipatory-utility frameworks propose that such attenuated precision on reward-error signals may reduce anticipatory pleasure and motivational drive (35). This provides a computational route to i.e., anhedonia and avolition.

At the neurobiological level, this pathology of precision-weighting has been linked to dysregulation in both glutamatergic and GABAergic systems. NMDA receptor dysfunction, which is implicated in schizophrenia, affects synaptic gain and thus the encoding of prediction error signals (26). Complementing this, GABAergic abnormalities-evidenced by decreased occipital GABA concentrations in patients-further disrupt the excitation-inhibition balance crucial for appropriate precision-weighting (36). These neurochemical imbalances manifest in circuit-level changes: individuals exhibit reduced visually induced gamma oscillation frequencies and impaired orientation discrimination, both linked to excitation–inhibition imbalances in visual cortex.

Empirical studies using tasks like oddball detection provide further evidence for this framework. Individuals with schizophrenia often show reduced mismatch negativity responses, consistent with improper error signalling (37). DCM analyses reveal the circuit-level consequences of these abnormalities: individuals show diminished local synaptic connectivity, particularly between inhibitory interneurons and superficial pyramidal cells (the putative error units in predictive coding), with connectivity deficits correlating with negative symptom severity (36). Furthermore, DCM analyses have found weakened feedback connectivity in cortical hierarchies, supporting the notion of a breakdown in top-down predictive stability. Predictive-processing accounts also allow hallucinations to arise at different levels of the hierarchy and across sensory modalities. Aberrant precision may be expressed within modality-specific circuits (e.g., auditory or visual pathways) or at higher associative levels that impose overly precise priors. Such models also permit priors that bias perception toward unstructured noise or volatility, making genuine signals harder to identify (12, 38). In summary, schizophrenia can be cast as a disorder of belief updating: the filters that should correct false beliefs via error signals are themselves corrupted, leaving aberrant beliefs (paranoia, hallucinations) unchecked (38).

### 4.2 Autism: weak priors and sensory overweighting

Autism Spectrum Disorder (ASD) offers a contrasting case to schizophrenia within predictive coding accounts of cognition (39). Although autism is not classified as a psychiatric disorder, it is frequently seen in neurodevelopmental and mental health services, and computational models of inference have been used to understand autistic perception and cognition. The influential theory by Pellicano and Burr (40) posits that autistic individuals form unusually weak priors, and this has been extended into predictive coding accounts that highlight atypical inference processes in ASD (41). In Bayesian terms, the prior in autistic perception is assigned low precision, leading to perceptual hypersensitivity and a focus on raw input (42). In other words, the posterior estimate is driven primarily by the likelihood (sensory evidence), with minimal top-down constraint. In Bayesian terms, this corresponds to an inference regime in which posterior beliefs are dominated by sensory evidence because priors are assigned abnormally low precision.

Behaviourally, this manifests as an insistence on *sameness* and difficulty generalising from past experience (since each situation is processed afresh, without strongly applying past lessons). It also aligns with enhanced local processing versus impaired global integration (the autistic brain doesn’t use broad priors to “fill in” gaps) (40, 43).

Neurophysiologically, researchers have noted an excitation/inhibition imbalance in ASD cortical circuits (44, 45). One interpretation is that inhibitory processes that help implement predictions (by suppressing predictable inputs) are weaker, leading to a surfeit of unsuppressed (unexpected) signal. This may explain why many autistic individuals experience sensory overwhelm in environments with high levels of stimulation – their brains are not effectively filtering out predictable background information. Additionally, predictive coding accounts of autism highlight differences in precision modulation by neuromodulators like serotonin or acetylcholine, which may underlie the diminished influence of priors (46). Interestingly, the proposed circuit abnormalities in autism – involving reduced influence of feedback connections and altered gain control in superficial pyramidal cells – mirror those in schizophrenia but with different parameter settings, suggesting these conditions may represent opposing ends of a predictive coding spectrum (42). While the full picture is complex, the overarching view is that autism involves atypical inference-perception and learning that lean too heavily on the immediate evidence and struggle to incorporate the abstract, probabilistic regularities that typically guide human perception.

### 4.3 Depression: maladaptive attractor states and failure to update

**Figure 3:**
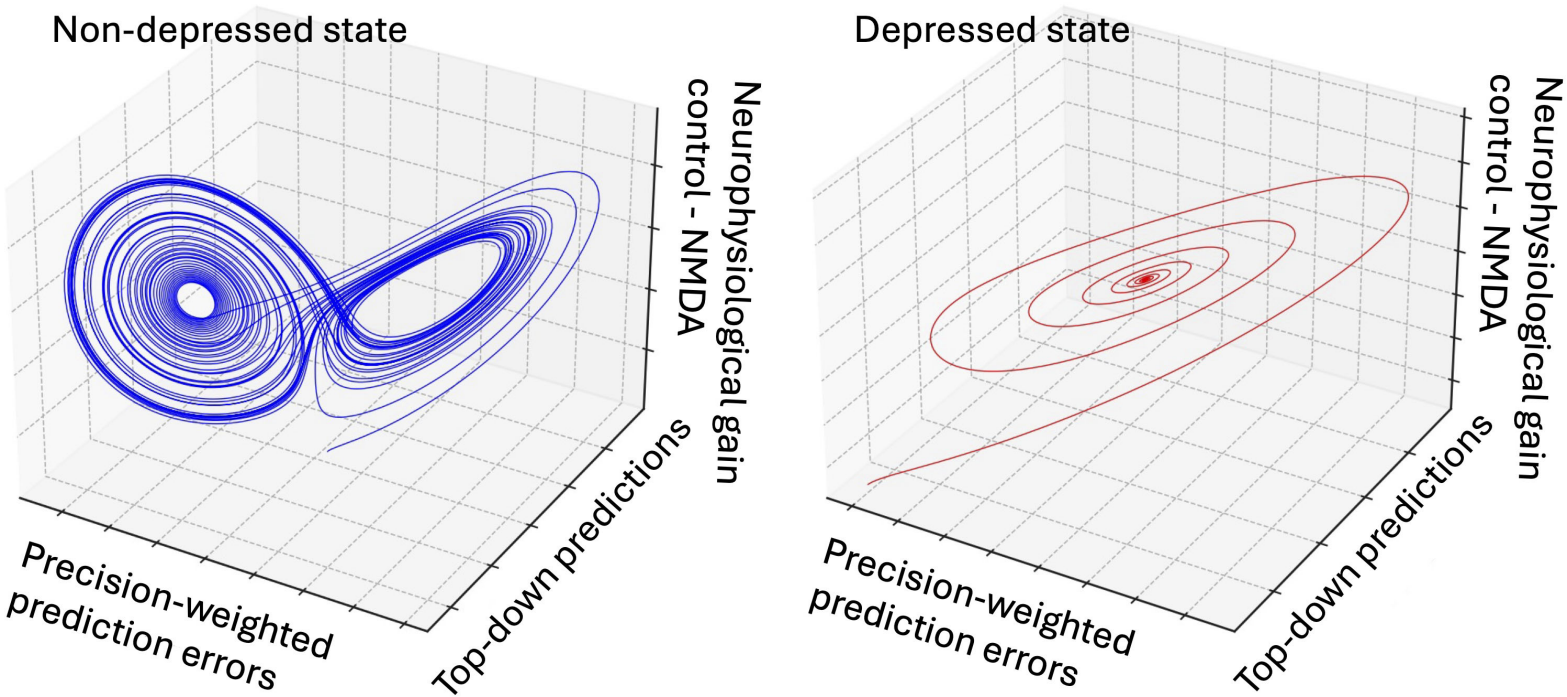
Conceptual illustration of differences in neural dynamics between healthy (leftpanel) and depressed (right panel) states using a generic attractor framework. The system shown is a toy dynamical model for intuition, not a realistic simulation of a particular brain circuit. The three axes represent key computational variables in predictive coding: X-axis: Precision-weighted prediction error (how much sensory input updates beliefs). Y-axis: Top-down priors (strength of internal expectations, including cognitive biases). Z-axis: Neuromodulatory gain control (influence of neurotransmitters like NMDA on precision). In the healthy state (blue attractor), neural dynamics are chaotic and flexible, allowing for continuous updating of beliefs in response to sensory input. The system explores a broad range of states, reflecting an adaptive balance between prior beliefs and prediction errors. In contrast, the depressed state (red attractor) exhibits a collapsed, deep attractor basin, indicative of rigid, maladaptive inference. Here, overweighted priors (high Y) dominate, while prediction error updates are suppressed (low X), leading to inflexible belief updating. Additionally, reduced neuromodulatory gain (low Z) diminishes the system’s ability to dynamically adjust sensory precision, mirroring reduced serotonergic and dopaminergic function in depression.

Major depression is often characterised by rigid negative beliefs and an inability to adapt to positive new information. In predictive coding terms, one can think of depression as the brain getting trapped in a maladaptive predictive model that it fails to update despite contrary evidence. From a dynamical systems perspective, this corresponds to a pathological attractor state in neural activity (47, 48). This dynamical picture is the state-space counterpart of precision-weighted prediction-error updates in predictive coding: stable attractors correspond to self-consistent posterior beliefs and behavioural policies that the system returns to after small perturbations. The brain’s state-space dynamics settle into a basin of low energy (a pessimistic, self-consistent model of the world) and exhibit reduced flexibility to escape that basin (Equation 6). Formally, we might write the neural state evolution as:

(6) $$\frac{dx}{dt}=f(x)$$

and say that f(x) has an attractor that represents a depressive state. Normally, prediction errors (surprising positive events, changes in environment) would perturb the system out of that attractor, leading to an update of beliefs (e.g. learning that things aren’t as hopeless as expected). In depression, however, there seems to be a failure to effectively minimise free energy with respect to new inputs. The brain’s model does not get updated by unexpected good news; instead, the system may downweight those prediction errors or interpret them in a way that conforms to the negative prior belief (49) (e.g. “it was a fluke,” “it won’t last”, see **Figure 3**). We present this attractor-based formulation as a conceptual synthesis of existing computational accounts (47–49), rather than as a claim that a specific low-dimensional dynamical system has been empirically identified in neural data. It is worth noting that several alternative predictive-processing accounts of depression emphasise different classes of priors and error signals. Some formulations highlight the role of interpersonal expectations and social threat sensitivity, in which depressive states can emerge from an evolutionarily conserved “better-safe-than-sorry” strategy that becomes maladaptive when overly precise or resistant to updating (50). More comprehensive reviews also outline additional variants, including models centred on aberrant precision control, biobehavioural threat monitoring, and dysregulated policy selection (51). Our attractor-based formulation should therefore be understood as one mechanistic perspective within this broader theoretical landscape.

Empirical evidence supports disrupted predictive processing in depression. For instance, depressed individuals show blunted neural responses to unexpected rewards (a kind of reduced reward prediction error signalling) (52). This is consistent with anhedonic depression, where people no longer feel interest or reward in things they had previously. On the circuit level, depression has been associated with impaired top-down connectivity and reduced neuromodulatory drive (e.g. serotonin), which could both contribute to a sluggish predictive coding system that does not revise its priors promptly (53).

Unlike the positive symptoms of schizophrenia (with its overweighted priors) or autism (with its under-weighted priors), depression may represent a different type of predictive coding dysfunction-one characterized by normal weighting but impaired updating dynamics. In the free-energy framework, one could say the depressive brain is not adequately exploring the space of alternative hypotheses; it is stuck with a high *model evidence* for a dark outlook because it isn’t sampling the environment in an unbiased way. This view also resonates with psychological observations like confirmation bias in depression (selectively attending to negative feedback). At the cognitive level, the negative schema described in cognitive therapy can be recast as a prior with excessive precision that resists updating. This perspective suggests specific therapeutic approaches. Treatments like behavioral activation, which deliberately exposes individuals to potentially rewarding activities (54), could work by forcing the sampling of environments that generate prediction errors contrary to the depressive model. Similarly, cognitive therapy may function by explicitly challenging the high-precision priors that maintain the depressive attractor state (55).

### 4.4 Obsessive–compulsive disorder: overactive error signals and precision

Obsessive–compulsive disorder can be understood as an exaggeration of the brain’s error signals and uncertainty responses. In OCD, patients are tortured by a feeling that *something is wrong* or incomplete, leading to repetitive behaviours to mitigate that anxiety. Computationally, this maps to excessive precision-weighting of prediction errors, especially in the realm of threat or danger predictions (56, 57).

(7) $$\frac{d\mu }{dt}=\epsilon \cdot \frac{\partial \hat{y}}{\partial \mu }$$

Equation 7 describes how internal beliefs μ) are updated based on prediction errors ( $\epsilon =y-\hat{y}$) and the sensitivity of predicted input $\hat{y}$ to those beliefs. In OCD, the precision assigned to ϵ becomes pathologically high, effectively amplifying small discrepancies and driving excessive belief updates, even when the external world offers reassurance (58).

Even when things are objectively fine, the OCD brain generates a strong error signal indicating a discrepancy (e.g. “maybe my hands are still not clean” or “the door isn’t truly locked”). These error signals are given abnormally high confidence, compelling the individual to act on them (wash again, check again). Essentially, the system has too low a threshold for declaring a prediction error and then cannot easily cancel that error signal.

One way to formalise this is to say that the complexity term in model optimisation dominates: the brain’s model remains extremely complex and unwilling to accept that a simpler explanation (e.g. “the stove is off and nothing bad will happen”) is sufficient. The hierarchy in predictive coding might get “stuck” with intermediate-level error units firing constantly. This interpretation is consistent with findings that OCD patients show an action-confidence dissociation driven by oversized unsigned prediction-error responses (59), which in predictive-processing terms reflects excessive precision on low-level error. This distinguishes OCD from other disorders we’ve discussed: OCD involves appropriate weighting but with excessive precision on specific categories of errors, particularly those related to safety, contamination, or moral concerns.

Neurobiologically, OCD has been strongly linked to hyperactivity in specific circuits like the cortico-striatal-thalamo-cortical (CSTC) loop, particularly involving the orbitofrontal cortex (OFC) and anterior cingulate cortex (ACC) (60, 61). These regions are associated with monitoring for errors and adjusting behaviour. Imaging studies show that at baseline and during symptom provocation, OFC and ACC are over-active in OCD patients, and successful treatment tends to normalise this hyperactivity. This aligns perfectly with a predictive coding account: the OFC/ACC could be considered hubs of computing whether outcomes match predictions (ACC signals “error/conflict,” OFC encodes expected value and violation). Their hyperactivity means the brain is constantly flagging potential errors or unexpected outcomes, even when unwarranted.

Computational models suggest that increasing the precision of certain prediction errors can produce OCD-like repetitive checking behaviour as the system tries to resolve an endless stream of perceived mismatches (56, 58). Furthermore, recent theoretical work proposes OCD as a disorder of hyper-attention to internal signals – effectively, too much attention (precision) is allocated to thoughts of potential disaster, and not enough to external evidence that things are okay.

### 4.5 Anxiety disorders: exaggerated uncertainty and threat predictions

Anxiety disorders, including generalised anxiety and panic disorder, can be viewed through predictive coding as disorders of threat inference under uncertainty. Anxious individuals often overestimate the likelihood of harm and have difficulty tolerating uncertainty. In predictive processing terms, there may be an over-precision (49, 62, 63) of negative predictions about the future, coupled with an inability to down-regulate error signals related to ambiguous stimuli. The result is a chronic state of expecting the worst (a strongly weighted prior of threat) and a hypersensitivity to any signal that might indicate danger (even if it’s equivocal).

One formal expression is that the estimated volatility of the environment is high – the brain assumes that unexpected bad events can happen at any time, thus it keeps prediction errors for threat cues high and doesn’t easily extinguish fear expectations. A simple model might add a noise term *w* to state transitions and treat it as very large in anxiety:

(8) $$x_{t+1}=f(x_{t})+\omega_{t}$$

with the anxious brain assuming large *w* (environmental uncertainty) in Equation 8. This leads to maintaining a state of vigilance as the optimal strategy. While sharing some features with OCD, anxiety disorders are distinct in their predictive coding profile. Where OCD involves excessive precision on specific error signals (e.g., “the door might not be locked”), anxiety disorders feature a more generalized overestimation of environmental volatility and threat probability. Different anxiety subtypes may represent variations within this framework:

generalized anxiety disorder involves broad overestimation of threat across multiple domains, panic disorder features catastrophic misinterpretations of bodily sensations as immediate threats, and specific phobias exhibit localized precision abnormalities for particular stimuli (20).

Neuroimaging findings in anxiety align with this picture. The amygdala, a key region for threat processing, shows heightened activation to uncertain or ambiguous cues in anxious individuals (64). Essentially, the amygdala responds with alarm even when a situation is only potentially aversive, reflecting a failure to attenuate prediction errors about threat in uncertain contexts. At the same time, top-down regulation from the prefrontal cortex (which in predictive coding would convey reassuring predictions or reappraisals) is often weaker in anxiety. This combination – strong bottom-up error signals for threat, weak top-down calming predictions – yields a dominance of “fear prediction errors” that sustain anxiety (65).

Clinically, this maps to phenomena like hyper-vigilance (constantly scanning for danger) and intolerance of uncertainty (distress when outcomes are unpredictable). In computational terms, one could say the prior for a safe outcome has abnormally low precision in anxious individuals; instead, the prior expectancy might be biased toward danger, and any deviation (even safe signals) fails to fully convince the system that all is well. Treatments like exposure therapy can be seen as attempts to recalibrate these prediction weights–by repeated safe exposures, the patient’s brain is encouraged to assign greater weight to the “I am safe” prediction relative to the “something bad will happen” prediction error (66).

### 4.6 Bipolar disorder: instability of precision and network dynamics

Bipolar disorder, marked by oscillations between manic (or hypo-manic) and depressive states, can be conceptualised in predictive coding terms as an instability in how the brain regulates precision across different states (47, 53). One proposal is that bipolar disorder involves difficulty maintaining a consistent hierarchical inference: the neurotransmitter and modulatory systems that set precision (like NMDA, dopamine and serotonin) fluctuate abnormally, causing the brain to over-fit at times and under-fit at others (67).

(9) $$\frac{d\mu }{dt}=\alpha (t)\cdot \epsilon \cdot \frac{\partial \hat{y}}{\partial \mu }$$

In Equation 9, μ denotes an internal belief or mood-related latent state, ϵ is the prediction error, and α(t) is a time varying precision or gain parameter (modulated by NMDA, dopamine or serotonin) (47, 53). In bipolar disorder, α(t) becomes dysregulated - increasing excessively in mania (leading to overconfident updates and reward seeking behaviour), and dropping in depression (blunting belief updates and reinforcing negative expectations). This instability pushes the system between maladaptive attractor states, resulting in oscillations in mood and behaviour.

In mania, the brain may assign excessive precision to active, exploratory policies and reward-predicting priors (“everything will turn out great”), leading to overconfidence, racing thoughts, and risk-taking (the internal model is too strongly believed). In depression (the other pole), the precision might crash for positive priors, leading to the state described earlier of hypo-learning and negativity (68, 69). Thus, the homeostatic control of precision weights fails to keep the system in balance, and it instead switches between attractor states of high vs. low confidence. One can imagine a double-well energy landscape for brain states – one basin corresponds to depressive mode, another to manic mode – and the system unpredictably jumps between them due to regulatory noise.

State-space models have been used to simulate such phenomena, where a parameter representing gain or arousal varies over time and pushes the system from one regime to another (1). For instance, a simplified model might have: when precision parameter *α* is above a threshold, the network engages a “manic” pattern of activity (high reward-seeking, low error sensitivity), and when *α* falls below a threshold, a “depressive” pattern emerges. Abrupt neuromodulatory shifts (say, in dopamine tone) could trigger these transitions.

Neuroimaging studies of bipolar disorder have indeed found differences in connectivity between mood states – e.g. mania is associated with heightened connectivity in reward circuits and reduced prefrontal oversight, whereas depression shows the opposite. There is evidence of dysregulated oscillatory activity and signalling between the prefrontal cortex and limbic regions (like the amygdala) in bipolar patients. In predictive coding terms, this might reflect inconsistent application of top-down constraints on emotional inference: sometimes too much (leading to an inflated, unfettered mood in mania) and sometimes too little (leading to depressive pessimism) (69, 70).

While bipolar disorder is complex and not as extensively modelled in predictive coding as other disorders, this perspective provides a coherent narrative: it is a disorder of regulatory oscillation, where the mechanisms that normally stabilise our predictive mind – keeping emotional predictions attuned to reality – themselves become unstable.

While our focus to this point has been on neurophysiological mechanisms, predictive-processing accounts of psychopathology also extend naturally to behaviour. Under active inference, actions are selected to minimise expected free energy, reducing uncertainty and maintaining preferred states (17). This means that characteristic behaviours across disorder (such as avoidance in anxiety, compulsions in OCD, or social withdrawal in depression) can be understood as policies that initially reduce volatility or prediction error but become maladaptive when they reinforce overly precise priors or limit access to corrective evidence (51).

## 5 Discussion: implications for psychiatry

The above examples illustrate how predictive coding can unify our understanding of diverse psychiatric symptoms under a common computational framework. This approach may have several broad implications for research and clinical practice, which we explore in the following subsections.

### 5.1 Mechanistic biomarkers

Embracing computational models opens the door to identifying biomarkers based on circuit function rather than just phenomenology (71–73). Instead of purely descriptive diagnoses, clinicians could measure specific deviations in a patient’s predictive coding dynamics (for example, an EEG marker of abnormal error signalling or a connectivity pattern from DCM) as a biomarker of illness. Such biomarkers would directly reflect underlying mechanisms – for instance, reduced top-down connectivity or heightened sensory precision –linking symptoms to neurobiology (31).

This is in line with the goals of precision psychiatry, which seeks objective, quantitative measures to classify and treat mental disorders. Computational psychiatry is seen as an essential tool in this effort, helping to translate between observed behaviour/neural data and the latent neurocomputational parameters that differ across individuals. Over time, a catalogue of predictive coding abnormalities (e.g. “hyper-precision of threat prediction” for certain anxiety disorders, or “NMDA hypofunction leading to reduced error correction” for certain psychoses) could form a basis for a new nosology grounded in mechanism. Importantly, these models can be iteratively refined and validated against longitudinal data, improving their reliability as biomarkers.

### 5.2 Personalised and precision treatment

A computational perspective can inform treatment by tailoring interventions to the patient’s specific predictive processing profile. For instance, two patients might both have anxiety, but one might show exaggerated bottom-up error signals (sensory hypervigilance) while another shows mainly a top-down prior bias (catastrophic thinking). These nuances could suggest different treatments (67): perhaps the former would benefit more from stimulus-driven desensitisation (to recalibrate error responses), whereas the latter might benefit from cognitive restructuring techniques (to adjust overly precise priors).

On the pharmacological side, if a model indicates that a patient’s symptoms stem from low precision in a certain circuit (implying maybe inadequate neuromodulatory drive), one might choose a drug that boosts that neuromodulator (1). In schizophrenia, for example, the predictive coding account implicates dopamine in precision control; treatments that restore dopamine balance (antipsychotics) can be understood as partially re-tuning precision weights to normal levels. More broadly, *in silico* modelling can simulate how a given patient’s brain might respond to different interventions. This aligns with the concept of personalised psychiatry, using a patient’s data in a model to predict the optimal treatment strategy. While still in early stages, such approaches could improve outcomes by moving beyond one-size-fits-all therapy toward individualised care plans based on computational phenotyping.

Along these lines, predictive coding also provides a framework upon which to integrate psychotherapy and behavioural treatments. With a given disorder or dysfunction described in biologically informed predictive coding terms it becomes possible to map the contribution of a drug (such as ketamine for depression) to alleviating depression (pharmacologically increasing sensitivity to prediction errors as well as imposing a more positive attractor state) and the usefulness of therapy to maximise this opportunity such as by working to imbed the “corrected” thinking to maintain the healthy state. Ketamine has indeed been shown to increase sensitivity to prediction error in the hours where the antidepressant state emerges (74). Antidepressant medicines have been shown to be most effective when combined with therapy.

### 5.3 Novel therapeutics and interventions

The predictive coding framework inspires new intervention approaches as well. One exciting area is computationally informed neurostimulation. For example, if OCD is conceptualised as a hyperactive error signal in ACC, treatments like deep brain stimulation (DBS) could be guided to specifically down-regulate that error unit activity. There are efforts to design closed-loop stimulation devices that use real-time recordings to adjust stimulation in response to abnormal neural patterns (like a burst of pathological error signalling). In theory, a closed-loop system could continuously drive the brain toward a lower free-energy state, essentially helping the patient’s brain to more effectively minimise prediction errors.

Another avenue is training paradigms or biofeedback: patients could be given tasks that implicitly re-balance their predictive coding (58). For instance, video games that reward the patient for reinterpreting 453 surprising cues might strengthen certain neural pathways for error processing. Moreover, the emphasis on active inference (the idea that the brain not only passively updates but also takes actions to fulfil predictions) suggests behavioural interventions could aim to break maladaptive active inference loops (75). For example, encouraging patients with depression to engage in new, surprising activities can provide prediction errors that force an update to their negative priors (essentially “shaking” the system out of the depressive attractor).

Finally, pharmacotherapy development can benefit from these models by targeting the identified circuit parameters: drugs that affect synaptic gain, adaptation, or oscillatory coupling might be tested in computational models of disorders before clinical trials, increasing the rationale for certain targets. In sum, predictive coding offers a principled framework to design interventions that steer the brain’s computations, whether through chemicals, devices, or behavioural experience, to restore healthy inference.

### 5.4 Challenges

Despite its promise, translating computational psychiatry from theory to clinical practice faces several key challenges. Foremost among them is the difficulty of integrating computational insights into clinical taxonomies and diagnostic systems in a way that allows for sensitive and specific identification of pathology.

One major issue is *co-morbidity*. For instance, autism and schizophrenia are often comorbid (76), yet they are frequently cited as archetypal examples of contrasting predictive coding mechanisms (42). Any clinically useful tool must be capable of detecting both conditions when they co-occur in the same individual. The fact that they can co-occur implies that predictive coding alterations in the brain may exhibit some degree of domain specificity, and any diagnostic framework must be designed accordingly.

Another challenge is the marked heterogeneity within diagnostic categories, particularly mood disorders. Two individuals may receive the same diagnosis-such as major depressive disorder or schizophrenia-yet share no overlapping symptoms. Anhedonia experienced in bipolar disorder has been found to have partially distinct neurophysiology to that in unipolar depression (77). Furthermore, diagnoses often evolve over time. A person initially diagnosed with major depressive disorder may later be reclassified as bipolar following the emergence of mania, and may subsequently transition from bipolar type II to type I. Similarly, an individual experiencing cyclical mood changes might initially receive a diagnosis of bipolar disorder, only to have it later revised to premenstrual dysphoric disorder when the temporal pattern of symptoms is recognised.

These examples highlight a critical point: any attempt to ground neurocomputational psychiatry in mechanistic frameworks must not inherit the foundational limitations of the The Diagnostic and Statistical Manual of Mental Disorders (DSM) (78). Diagnostic instability and symptom heterogeneity challenge the assumption that current categories map onto distinct underlying neurobiology.

Indeed, these limitations may help explain why biomarkers grounded in predictive coding-such as mis-match negativity-have not yet demonstrated sufficient specificity or reliability for clinical translation. This remains true whether such biomarkers are assessed using basic DCM or more conventional event related potential analyses. One potential solution is to initially validate these methods using enriched sampling strategies that focus on “ideal” or exemplar participants. However, for true clinical utility, these tools must ultimately prove robust in the messiness of real-world clinical populations.

These challenges, however, are not fatal to the feasibility of the approach. Although the DSM classifies mental disorders into discrete categories, treatments are often based on symptom domains, which themselves cut across diagnostic boundaries. For example, antipsychotic medications are used to treat psychosis regardless of whether it arises in the context of schizophrenia or bipolar disorder. Similarly, depressed mood across bipolar disorder, premenstrual dysphoric disorder, and major depressive disorder is commonly treated with selective serotonin reuptake inhibitors (SSRIs). While the choice and dosing of medication may be partially informed by overarching diagnostic labels, this is a far cry from the categorical logic of treating a viral infection with an antibiotic - a treatment that is not only ineffective, but biologically inappropriate and without mechanistic rationale. Optimistically speaking, predictive coding may support the development of precision psychiatric medicine meaning optimal choices may be based upon an individuals neurophysiology as well as symptoms (again rather than DSM classification).

In this light, predictive coding may find its greatest utility not in redefining diagnostic categories, but in providing mechanistic descriptions of symptom classes-dimensions of dysfunction that are either necessary for a diagnosis or that support meaningful sub-classification within a diagnosis. Between sub-classifications or even individuals it may support the selection of mechanistically distinct treatments (such as ketamine over and SSRI for depression). Such models offer hypotheses that are mathematically constrained and amenable to empirical testing via neurophysiology.

A related limitation concerns the specificity of predictive-processing accounts themselves. Many of the core quantities invoked by predictive coding (such as prediction errors, value updating, and precision) also arise in alternative computational frameworks including reinforcement learning, Bayesian decision theory, and control-theoretic models (79). As a consequence, the same behavioural or neural phenomena can often be explained within multiple formalisms, limiting the discriminative power of predictive coding unless models are formulated to make distinct empirical predictions. Developing such adjudicable predictions remains an important direction for future work.

Another major challenge is ensuring that the computational parameters used in these models can be reliably estimated in individual patients using accessible technologies. Advances in electroencephalography (EEG), including increased portability and affordability, offer a promising path forward. Similarly, developments in magnetoencephalography (MEG), particularly the use of optically pumped magnetometers, are making high-resolution, non-invasive brain measurements more feasible in clinical contexts. Surface encephalography, in particular, provides the most accessible, non-invasive route to probing neural circuit dynamics at the level inferred by neural mass and mean field models.

A final challenge concerns the need for longitudinal validation. For computational biomarkers to be clinically useful, their stability and predictive value over time must be established. Few studies have examined the test-retest reliability of DCM-based biomarkers or the consistency of individual parameter “fingerprints” across multiple sessions.

## 6 Conclusion

Predictive coding has quickly become a leading theoretical framework in cognitive and computational neuroscience, and shows great promise in psychiatry. By viewing mental disorders as disturbances in the brain’s predictive machinery, we gain a common language to describe phenomena as varied as hallucinations, anxiety, and cognitive inflexibility. This approach encourages researchers and clinicians to think in terms of circuits and computations - how is the brain weighting its predictions vs. errors? which connections are failing to convey predictions? - rather than solely in terms of symptoms and subjective reports. The advantage of such a framework is not only its explanatory power, but also its generality: it leads to testable hypotheses and models that can be validated with neural data. As we refine these models (through approaches like DCM, neural mass modelling, and machine learning on large-scale data), we move closer to identifying the true underlying dimensions of psychopathology that cut across traditional diagnoses.

## Data Availability

The original contributions presented in the study are included in the article/supplementary material. Further inquiries can be directed to the corresponding author.

## References

[1] Montague R Dolan RJ Friston KJ Dayan P . Computational psychiatry. Trends Cogn Sci. (2012) 16:72–80. doi: 10.1016/j.tics.2011.11.018, PMID: 22177032 PMC3556822

[2] Adams RA Stephan KE Brown HR Frith CD Friston KJ . The computational anatomy of psychosis. Front Psychiatry. (2013) 4:47. doi: 10.3389/fpsyt.2013.00047, PMID: 23750138 PMC3667557

[3] Petzschner FH Weber LAE Gard T Stephan KE . Computational psychosomatics and computational psychiatry: Toward a joint framework for differential diagnosis. Biol Psychiatry. (2017) 82:421–30. doi: 10.1016/j.biopsych.2017.05.012, PMID: 28619481

[4] Gauld C Dumas G Fakra Éric Mattout Jérémie Micoulaud-Franchi J-A . Les trois cultures de la psychiatrie computationnelle. Annales Médico-psychologiques Rev psychiatrique. (2021) 179:63–71. doi: 10.1016/j.amp.2020.11.011 PMC7687400 33250519

[5] Qela B Damiani S Santis SDe Groppi F Pichiecchio A Asteggiano C . Predictive coding in neuropsychiatric disorders: A systematic transdiagnostic review. Neurosci Biobehav Rev. (2025) 169:106020. doi: 10.1016/j.neubiorev.2025.106020, PMID: 39828236

[6] Adams RA Huys QJM Roiser JP . Computational psychiatry: towards a mathematically informed understanding of mental illness. J Neurol Neurosurgery Psychiatry. (2016) 87:53–63. doi: 10.1136/jnnp-2015-310737, PMID: 26157034 PMC4717449

[7] Friston K . A theory of cortical responses. Philos Trans R Soc B. (2005) 360:815–36. doi: 10.1098/rstb.2005.1622, PMID: 15937014 PMC1569488

[8] Clark A . Whatever next? predictive brains, situated agents, and the future of cognitive science. Behav Brain Sci. (2013) 36:181–204. doi: 10.1017/S0140525X12000477, PMID: 23663408

[9] Hodson R Mehta M Smith R . The empirical status of predictive coding and active inference. Neurosci Biobehav Rev. (2024) 157:105473. doi: 10.1016/j.neubiorev.2023.105473, PMID: 38030100

[10] Friston K . The free-energy principle: a unified brain theory? Nat Rev Neurosci. (2010) 11:127–38. doi: 10.1038/nrn2787, PMID: 20068583

[11] Friston K Stephan KE . Free-energy and the brain. Synthese. (2007) 159:417–58. doi: 10.1007/s11229-007-9237-y, PMID: 19325932 PMC2660582

[12] Sterzer P Adams RA Fletcher P Frith C Lawrie SM Muckli L . The predictive coding account of psychosis. Biol Psychiatry. (2018) 84:634–43. doi: 10.1016/j.biopsych.2018.05.015, PMID: 30007575 PMC6169400

[13] Friston KJ Harrison L Penny W . Dynamic causal modelling. NeuroImage. (2003) 19:1273–302. doi: 10.1016/S1053-8119(03)00202-7, PMID: 12948688

[14] Knill DC Pouget A . The bayesian brain: the role of uncertainty in neural coding and computation. Trends Neurosci. (2004) 27:712–9. doi: 10.1016/j.tins.2004.10.007, PMID: 15541511

[15] Rao RPN Ballard DH . Predictive coding in the visual cortex: a functional interpretation of some extra-classical receptive-field effects. Nat Neurosci. (1999) 2:79–87. doi: 10.1038/4580, PMID: 10195184

[16] Hohwy J . The Predictive Mind. Oxford, UK: Oxford University Press (2013).

[17] Friston K FitzGerald T Rigoli F Schwartenbeck P Pezzulo G . Active inference: a process theory. Neural Comput. (2017) 29:1–49. doi: 10.1162/NECO_a_00912, PMID: 27870614

[18] Deco G Jirsa V McIntosh AR . The dynamic brain: from spiking neurons to neural masses and cortical fields. PloS Comput Biol. (2008) 4:e1000092. doi: 10.1371/journal.pcbi.1000092, PMID: 18769680 PMC2519166

[19] Breakspear M . Dynamic models of large-scale brain activity. Nat Neurosci. (2017) 20:340–52. doi: 10.1038/nn.4497, PMID: 28230845

[20] Bastos AM Usrey WM Adams RA Mangun GR Fries P Friston KJ . Canonical microcircuits for predictive coding. Neuron. (2012) 76:695–711. doi: 10.1016/j.neuron.2012.10.038, PMID: 23177956 PMC3777738

[21] Moran RJ Pinotsis DA Friston K . Neural masses and fields in dynamic causal modeling. Front Comput Neurosci. (2013) 7:57. doi: 10.3389/fncom.2013.00057, PMID: 23755005 PMC3664834

[22] Jansen BH Rit VG . Electroencephalogram and visual evoked potential generation in a mathematical model of coupled cortical columns. Biol Cybernetics. (1995) 73:357–66. doi: 10.1007/BF00199471, PMID: 7578475

[23] Hodgkin AL Huxley AF . A quantitative description of membrane current and its application to conduction and excitation in nerve. J Physiol. (1952) 117:500–44. doi: 10.1113/jphysiol.1952.sp004764, PMID: 12991237 PMC1392413

[24] Moran RJ Stephan KE Seidenbecher T Pape H-C Dolan RJ Friston KJ . Consistent spectral predictors for dynamic causal models of steady-state responses. NeuroImage. (2011) 55:1694–708. doi: 10.1016/j.neuroimage.2011.01.012, PMID: 21238593 PMC3093618

[25] Stephan KE Friston KJ Frith CD . Dysconnection in schizophrenia: from abnormal synaptic plasticity to failures of self-monitoring. Schizophr Bull. (2008) 35:509–27. doi: 10.1093/schbul/sbn176, PMID: 19155345 PMC2669579

[26] Berndt LCS Singh KD Shaw AD . Restoring synaptic balance in schizophrenia: Insights from a thalamo-cortical conductance-based model. bioRxiv Schizophrenia Bulletin. (2025). doi: 10.1093/schbul/sbaf149, PMID: 40931590 PMC13391648

[27] Fleming SM . Metacognition and confidence: A review and synthesis. Annu Rev Psychol. (2024) 75:241–68. doi: 10.1146/annurev-psych-022423-032425, PMID: 37722748

[28] Markov NT Vezoli J Chameau P Falchier A Quilodran R Huissoud C . Anatomy of hierarchy: feedforward and feedback pathways in macaque visual cortex. J Comp Neurol. (2014) 522:225–59. doi: 10.1002/cne.23458, PMID: 23983048 PMC4255240

[29] Murray Sherman S Guillery RW . Functional connections of cortical areas: A new view from the thalamus. Cambridge, MA: MIT Press (2013).

[30] Friston KJ Preller KH Mathys CD Cagnan H Heinzle J Razi A . Dynamic causal modelling revisited. NeuroImage. (2019) 199:730–44. doi: 10.1016/j.neuroimage.2017.02.045, PMID: 28219774 PMC6693530

[31] Friston K Redish AD Gordon JA . Computational psychiatry: the brain as a phantastic organ. Lancet Psychiatry. (2014) 1:148–58. doi: 10.1016/S2215-0366(14)70275-5, PMID: 26360579

[32] Corlett PR Mollick JA Kober H McCarthy C Hu J Thompson A . Hallucinations and strong priors. Trends Cogn Sci. (2019) 23:114–27. doi: 10.1016/j.tics.2018.12.001, PMID: 30583945 PMC6368358

[33] Stephan KE Mathys C . Computational approaches to psychiatry. Curr Opin Neurobiol. (2014) 25:85–92. doi: 10.1016/j.conb.2013.12.007, PMID: 24709605

[34] Corlett PR Honey GD Fletcher PC . Prediction error, ketamine and psychosis: An updated model. J Psychopharmacol. (2016) 30:1145–55. doi: 10.1177/0269881116650087, PMID: 27226342 PMC5105325

[35] Sepúlveda P Aitsahalia I Kumar K Atkin T Iigaya K . Addressing altered anticipation as a transdiagnostic target through computational psychiatry. Biol Psychiatry: Cogn Neurosci Neuroimaging. (2025) 9. doi: 10.1016/j.bpsc.2025.02.014, PMID: 40058458 PMC12353521

[36] Shaw AD Knight L Freeman TCA Williams GM Moran RJ Friston KJ . Oscillatory, computational, and behavioral evidence for impaired gabaergic inhibition in schizophrenia. Schizophr Bull. (2020) 46:345–53. doi: 10.1093/schbul/sbz066, PMID: 31219602 PMC7442335

[37] Umbricht D Krljes S . Mismatch negativity in schizophrenia: a meta-analysis. Schizophr Res. (2005) 76:1–23. doi: 10.1016/j.schres.2004.12.002, PMID: 15927795

[38] Powers AR Mathys C Corlett PR . Pavlovian conditioning–induced hallucinations result from overweighting of perceptual priors. Science. (2017) 357:596–600. doi: 10.1126/science.aan3458, PMID: 28798131 PMC5802347

[39] Seth AK Friston KJ . Active interoceptive inference and the emotional brain. Philos Trans R Soc B. (2016) 371:20160007. doi: 10.1098/rstb.2016.0007, PMID: 28080966 PMC5062097

[40] Pellicano E Burr D . When the world becomes ‘too real’: a bayesian explanation of autistic perception. Trends Cogn Sci. (2012) 16:504–10. doi: 10.1016/j.tics.2012.08.009, PMID: 22959875

[41] Cruys SVde Evers K Hallen Rvd Eylen LV Boets B Wit Lde . Precise minds in uncertain worlds: predictive coding in autism. psychol Rev. (2014) 121:649–75. doi: 10.1037/a0037665, PMID: 25347312

[42] Lawson RP Rees G Friston KJ . An aberrant precision account of autism. Front Hum Neurosci. (2014) 8:302. doi: 10.3389/fnhum.2014.00302, PMID: 24860482 PMC4030191

[43] Happé F Frith U . The weak coherence account: detail-focused cognitive style in autism spectrum disorders. J Autism Dev Disord. (2006) 36:5–25. doi: 10.1007/s10803-005-0039-0, PMID: 16450045

[44] Rubenstein JLR Merzenich MM . Model of autism: increased ratio of excitation/inhibition in key neural systems. Genes Brain Behav. (2003) 2:255–67. doi: 10.1034/j.1601-183X.2003.00037.x, PMID: 14606691 PMC6748642

[45] Sohal VS Rubenstein JLR . Excitatory–inhibitory balance as a framework for investigating mechanisms in neuropsychiatric disorders. Mol Psychiatry. (2019) 24:1248–57. doi: 10.1038/s41380-019-0426-0, PMID: 31089192 PMC6742424

[46] Kanai R Komura Y Shipp S Friston K . Cerebral hierarchies: predictive processing, precision and the pulvinar. Philos Trans R Soc B. (2015) 370:20140169. doi: 10.1098/rstb.2014.0169, PMID: 25823866 PMC4387510

[47] Huys QJM Daw ND Dayan P . Depression: a decision-theoretic analysis. Annu Rev Neurosci. (2015) 38:1–23. doi: 10.1146/annurev-neuro-071714-033928, PMID: 25705929

[48] Kube T . Biased belief updating in depression. Clin Psychol Rev. (2023) 103:102298. doi: 10.1016/j.cpr.2023.102298, PMID: 37290245

[49] Pulcu E Browning M . A misestimation of uncertainty in affective disorders. psychol Med. (2019) 49:403–11. doi: 10.1016/j.tics.2019.07.007, PMID: 31431340

[50] Badcock PB Davey CG Whittle S Allen NB Friston KJ . The depressed brain: An evolutionary systems theory. Trends Cogn Sci. (2017) 21:182–94. doi: 10.1016/j.tics.2017.01.005, PMID: 28161288

[51] Smith R Badcock P Friston KJ . Recent advances in the application of predictive coding and active inference models within clinical neuroscience. Psychiatry Clin Neurosci. (2021) 75:3–13. doi: 10.1111/pcn.13138, PMID: 32860285

[52] Rutledge RB Skandali N Dayan P Dolan RJ . Association of neural and emotional impacts of reward prediction errors with major depression. JAMA Psychiatry. (2017) 74:790–7. doi: 10.1001/jamapsychiatry.2017.1713, PMID: 28678984 PMC5710549

[53] Dayan P Huys QJM . Serotonin, inhibition, and negative mood. PloS Comput Biol. (2008) 4:e4. doi: 10.1371/journal.pcbi.0040004, PMID: 18248087 PMC2222921

[54] Jacobson NS Martell CR Dimidjian S . Behavioral activation treatment for depression: returning to contextual roots. Clin Psychol: Sci Pract. (2001) 8 255. doi: 10.1093/clipsy.8.3.255

[55] Beck AT . Cognitive therapy and the emotional disorders. New York, NY: Penguin (1979).

[56] Fradkin I Adams RA Parr T Roiser JP Huppert JD . Searching for an anchor in an unpredictable world: A computational model of obsessive-compulsive disorder. psychol Rev. (2020) 127:672–99. doi: 10.1037/rev0000188, PMID: 32105115

[57] Zhao Y-J Zhang Y Wang Q Manssuer L Cui H Ding Q . Evidence accumulation and neural correlates of uncertainty in obsessive-compulsive disorder. Biol Psychiatry: Cogn Neurosci Neuroimaging. (2023) 8:1058–65. doi: 10.1016/j.bpsc.2023.05.011, PMID: 37343660 PMC10555851

[58] Friston K Samothrakis S Mon R . Active inference and agency: optimal control without cost functions. Biol Cybernetics. (2012) 106:523–41. doi: 10.1007/s00422-012-0512-8, PMID: 22864468

[59] Vaghi MM Luyckx F Sule A Fineberg NA Robbins TW Martino BDe . Compulsivity reveals a novel dissociation between action and confidence. Neuron. (2017) 96:348–54. doi: 10.1016/j.neuron.2017.09.006, PMID: 28965997 PMC5643443

[60] Tang W Zhu Q Gong X Zhu C Wang Y Chen S . Cortico-striato-thalamo-cortical circuit abnormalities in obsessive-compulsive disorder: A voxel-based morphometric and fmri study of the whole brain. Behav Brain Res. (2016) 313:17–22. doi: 10.1016/j.bbr.2016.07.004, PMID: 27388149

[61] Gillan CM Robbins TW . Goal-directed learning and obsessive–compulsive disorder. Philosological Trans R Soc B. (2014) 369:20130475. doi: 10.1098/rstb.2013.0475, PMID: 25267818 PMC4186229

[62] Browning M Behrens TEJ Jocham G O’Reilly JX Bishop SJ . Anxious individuals have difficulty learning the causal statistics of aversive environments. Nat Neurosci. (2015) 18:590–6. doi: 10.1038/nn.3961, PMID: 25730669 PMC4644067

[63] Paulus MP Yu AJ . Emotion and decision-making: affect-driven belief systems in anxiety and depression. Trends Cogn Sci. (2012) 16:476–83. doi: 10.1016/j.tics.2012.07.009, PMID: 22898207 PMC3446252

[64] Grupe DW Nitschke JB . Uncertainty and anticipation in anxiety: an integrated neurobiological and psychological perspective. Nat Rev Neurosci. (2013) 14:488–501. doi: 10.1038/nrn3524, PMID: 23783199 PMC4276319

[65] Etkin A Wager TD . Functional neuroimaging of anxiety: a meta-analysis of emotional processing in ptsd, social anxiety disorder, and specific phobia. Am J Psychiatry. (2007) 164:1476–88. doi: 10.1176/appi.ajp.2007.07030504, PMID: 17898336 PMC3318959

[66] Nair A Rutledge RB Mason L . Under the hood: Using computational psychiatry to make psychological therapies more mechanism-focused. Front Psychiatry. (2020) 11:140. doi: 10.3389/fpsyt.2020.00140, PMID: 32256395 PMC7093344

[67] Williams LM . Precision psychiatry: a neural circuit taxonomy for depression and anxiety. Lancet Psychiatry. (2016) 3:472–80. doi: 10.1016/S2215-0366(15)00579-9, PMID: 27150382 PMC4922884

[68] Nguyen T-TT Kovacevic S Dev SI Lu K-C Liu TT Eyler LT . Dynamic functional connectivity in bipolar disorder is associated with executive function and processing speed: A preliminary study. Neuropsychology. (2017) 31:73–83. doi: 10.1037/neu0000317, PMID: 27775400 PMC5471616

[69] Phillips ML Swartz HA . A critical appraisal of neuro imaging studies of bipolar disorder: toward a new conceptualization of mood disorders. Biol Psychiatry. (2014) 75:434–40. doi: 10.1176/appi.ajp.2014.13081008, PMID: 24626773 PMC4119497

[70] Kim D-J Bolbecker AR Howell J Rass O Sporns O Hetrick WP . Disturbed resting state eeg synchronization in bipolar disorder: A graph-theoretic analysis. NeuroImage: Clin. (2013) 2:414–23. doi: 10.1016/j.nicl.2013.03.007, PMID: 24179795 PMC3777715

[71] Stephan KE Schlagenhauf F Huys QJM Raman S Deserno L Heinz A . Translational perspectives for computational neuroimaging. Neuron. (2015) 87:716–32. doi: 10.1016/j.neuron.2015.07.008, PMID: 26291157

[72] Huys QJM Maia TV Frank MJ . Computational psychiatry as a bridge from neuroscience to clinical applications. Nat Neurosci. (2016) 19:404–13. doi: 10.1038/nn.4238, PMID: 26906507 PMC5443409

[73] Frässle S Yao Y Schöbi D Aponte E Heinzle J Stephan KE . Generative models for clinical applications in computational psychiatry. Wiley Interdiscip Reviews: Cogn Sci. (2022) 13:e1560. 10.1002/wcs.1460 29369526

[74] Sumner RL McMillan R Spriggs MJ Campbell D Malpas G Maxwell E . Ketamine improves short-term plasticity in depression by enhancing sensitivity to prediction errors. Eur Neuropsychopharmacol. (2020) 38:73–85. doi: 10.1016/j.euroneuro.2020.07.009, PMID: 32763021

[75] Parr T Pezzulo G Friston KJ . Active inference: the free energy principle in mind, brain, and behavior. Cambridge, MA: MIT Press (2022).

[76] Lai M-C Kassee C Besney R Bonato S Hull L Mandy W . Prevalence of co-occurring mental health diagnoses in the autism population: a systematic review and meta-analysis. Lancet Psychiatry. (2019) 6:819–29. doi: 10.1016/S2215-0366(19)30289-5, PMID: 31447415

[77] Whitton AE Pizzagalli DA . Anhedonia in Depression and Bipolar Disorder. Cham: Springer International Publishing (2022) p. 111–27. 10.1007/7854_2022_323 PMC12665610 35397065

[78] American Psychiatric Association . Diagnostic and statistical manual of mental disorders. 5th ed. Washington, DC: American Psychiatric Publishing (2022).

[79] Kwisthout J Bekkering H Rooij IV . To be precise, the details don’t matter: On predictive processing, precision, and level of detail of predictions. Brain Cogn. (2017) 112:84–91. doi: 10.1016/j.bandc.2016.02.008, PMID: 27114040

